# Bibliometric Analysis of Research Hotspots and Emerging Trends in Mitochondrial DNA and Atherosclerosis (2004–2025)

**DOI:** 10.1155/crp/2121810

**Published:** 2026-06-21

**Authors:** Yun Ouyang, Gesheng Wang, Ming Zhang, Puxuan Min, Mengxiong Guo

**Affiliations:** ^1^ Graduate School, Beijing University of Chinese Medicine, Beijing, China, bucm.edu.cn; ^2^ Department of Neurology III, Dongfang Hospital, Beijing University of Chinese Medicine, Beijing, China, bucm.edu.cn

**Keywords:** atherosclerosis_1_, bibliometrics_5_, CiteSpace_4_, mitochondrial DNA_2_, oxidative stress_3_

## Abstract

**Objective:**

To explore the research course, hotspots, and development trends of mitochondrial DNA (mtDNA) and atherosclerosis (AS) based on knowledge graph technology, providing references for clinical and basic research in this field.

**Methods:**

Publications themed on mtDNA and AS published between 2004 and 2025 were retrieved from the Web of Science Core Collection (WOSCC) database. CiteSpace 6.4. R1 Advanced software was used to construct and visually analyze knowledge graphs of authors, institutions, keywords, etc.

**Results:**

A total of 341 publications were included, with the annual publication volume showing an overall upward trend. Research methods for mtDNA and AS are diversifying, with mechanistic investigations showing a trending focus toward specific molecular pathways. A total of 241 authors and 353 publishing institutions were analyzed. Ten keyword clusters were identified, including Cluster #0 oxidative stress, Cluster #1 mitochondrial DNA, Cluster #2 degradation, Cluster #3 atherosclerosis, Cluster #4 hydrogen peroxide, Cluster #5 roles, Cluster #6 MAPK pathway, Cluster #7 epigallocatechin gallate, Cluster #8 mitochondrial DNA copy number, and Cluster #9 vascular disease.

**Conclusion:**

Research has evolved from foundational mechanistic studies to a focus on translational and clinical applications. Moving forward, efforts should be intensified to enhance collaboration among research teams and deepen mechanistic investigations, thereby laying a solid foundation for clinical translation.

## 1. Introduction

Atherosclerosis (AS), a chronic inflammatory disease characterized by lipid accumulation and plaque formation in arterial walls, remains a leading cause of cardiovascular morbidity and mortality worldwide [1–6]. Statin therapy to control elevated low‐density lipoprotein cholesterol (LDL‐C) is currently the core approach for treating AS [1]. However, side effects of statins—such as muscle pain, liver damage, and increased risk of diabetes—often lead to intolerance and poor medication adherence among high‐risk cardiovascular disease (CVD) patients [7]. Therefore, exploring the pathogenesis of AS and identifying better therapeutic strategies is imperative.

Mitochondria are the energy engines of the human body [8]. As early as 1970, Greenberg [9] and colleagues first linked mitochondrial function to AS. Mitochondrial DNA (mtDNA) has emerged as a critical player in the pathogenesis of AS. On the one hand, oxidized low‐density lipoprotein (ox‐LDL) can induce the production of reactive oxygen species (ROS), leading to mtDNA damage. This damaged mtDNA then activates the TLR9 inflammatory signal and exacerbates the atherosclerotic inflammatory response [10]. On the other hand, under inflammatory conditions, vascular cell adhesion molecule 1 (VCAM‐1) can activate STING‐mediated inflammatory responses, thereby exacerbating AS [11]. mtDNA damage levels are also elevated in human atherosclerotic plaques and circulating leukocytes, which are closely associated with high‐risk plaques [12, 13].

While a large body of research has explored the multifaceted roles of mtDNA in AS, a comprehensive, systematic overview of the field’s evolution over the past 2 decades is lacking. Previous reviews have focused on specific mechanisms or clinical applications, but few have quantitatively mapped the global publication trends, core themes, and emerging frontiers. Bibliometric is a quantitative statistical method for analyzing published papers and their related data [14].

This bibliometric analysis fills a gap by examining 21 years of research (2004–2025). The 2004–2025 timeframe was selected because it coincides with the publication of landmark studies that established that mtDNA damage and mutations act as a direct trigger for oxidative stress–induced apoptosis and dysfunction in vascular endothelial cells [15]. This period captures the full arc of the discipline, from its foundational phase to its current expansion into translational and clinical research.

Our study aims to (1) characterize the growth and geographic distribution of mtDNA‐related AS research; (2) identify core research themes and their evolution over time; (3) distinguish well‐established topics from genuinely emerging directions; and (4) assess how bibliometric patterns align with key mechanistic and translational advances in the field. By providing a data‐driven synthesis, we aim to guide future research priorities and accelerate the translation of mtDNA‐targeted strategies into clinical practice for the prevention and treatment of AS.

## 2. Methods

### 2.1. Data Source and Search Strategy

We conducted a systematic search of the Web of Science Core Collection (WOSCC) database (https://www.webofscience com), which is widely recognized as a gold standard for bibliometric analyses in cardiovascular and molecular biology research. The search query was formulated as follows: “TS = (Mitochondrial DNA OR mtDNA) AND (atherosclerosis OR atheroscleroses OR atherogenesis),” with the retrieval period set from January 1, 2004, to December 31, 2025. The inclusion criteria were limited to “article,” the language as “English,” with the aim of selecting a specific subject and study purpose while also standardizing the language for analysis in the follow. Therefore, we restricted the Publications search time frame to 2004–2025. All retrieved publications were independently evaluated by two reviewers based on titles, abstracts, and keywords to exclude studies irrelevant to mtDNA and AS. In case of discrepancies between the two reviewers, a third reviewer made the final decision. To minimize biases caused by database updates, the Publications retrieval was completed within a single day (April 15, 2026).

### 2.2. Inclusion and Exclusion Criteria

Inclusion criteria are as follows: (1) Studies focusing on the role of mtDNA in the pathogenesis, progression, or treatment of AS in human or preclinical models and (2) studies published in English to ensure consistency in data extraction and analysis. Exclusion criteria are as follows: (1) Conference abstracts, book chapters, editorials, and errata, as these typically do not represent complete, peer‐reviewed findings; (2) studies unrelated to mtDNA or AS, as determined by title and abstract screening; and (3) duplicate publications of the same study, identified by comparing authors, title, and publication date.

### 2.3. Screening Results

**FIGURE 1:**
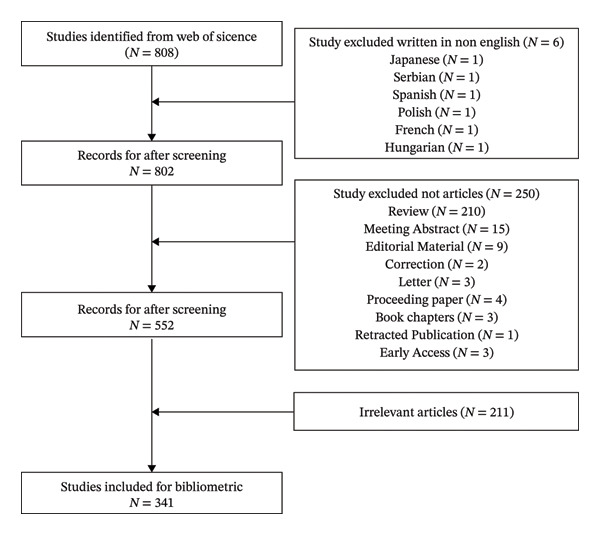
Literature search strategy.

A total of 808 publications were retrieved from the WOSCC database. Among the 808 retrieved records, 6 non‐English‐language articles were excluded, 250 nonoriginal research records (including reviews, meeting abstracts, and editorials) were removed, and 211 irrelevant articles were excluded after title/abstract screening, resulting in a final sample of 341 eligible publications included in this bibliometric analysis (Figure 1).

### 2.4. Data Collection and Analysis

Two researchers independently screened the publications in accordance with the inclusion and exclusion criteria. In case of discrepancies, a third researcher was consulted to reach a final decision. Data were exported from WOSCC in plain text format and imported into CiteSpace V. 6.4. R1 and VOSviewer 1.6. 20 for bibliometric analysis. Ultimately, 341 publications were included for visual analysis. The time interval was set from 2004 to 2025 with 1‐year slices, Top *N* = 200, and noncore parameters were maintained at the software’s default settings.

#### 2.4.1. Keyword Normalization

To eliminate bias caused by synonymy, abbreviations, and spelling variants, we performed standardized preprocessing of all keywords.

Synonym merging: Using CiteSpace’s built‐in “Alias” function combined with manual verification, we merged synonymous terms, including mitochondrial DNA/mtDNA/mitochondrial genome; atherosclerosis/AS/atheroscleroses/arteriosclerosis; vascular smooth muscle cell/VSMC; and oxidative stress/oxidative injury.

Stopword removal: We excluded noninformative general terms (e.g., study, analysis, research, and patient) that did not reflect specific research themes.

Spelling correction: We unified variant spellings (e.g., oxidative stress induced to oxidative stress–induced) to ensure analysis consistency.

#### 2.4.2. Network Pruning Method

To comprehensively capture the full topological structure of the keyword co‐occurrence network and present the global knowledge landscape of mtDNA and AS research, no network pruning strategy was applied (Pruning: None) in the present study. This approach retains all co‐occurrence relationships between keywords, allowing a transparent and objective visualization of the entire collaborative network and facilitating the identification of core themes and potential knowledge evolution.

#### 2.4.3. Cluster Quality Metrics

The reliability of the clustering results was evaluated using two core, peer‐recognized metrics.

Modularity *Q* (Q value): Quantifies the distinctness of network community structure. A Q value > 0.3 indicates a significant, nonrandom clustering structure. In this study, the modularity *Q* was 0.483, confirming a clear and meaningful thematic division of the keyword network.

Mean Silhouette Score (S value): Measures the internal homogeneity of each cluster. An S value > 0.5 indicates acceptable cluster consistency, while an S value > 0.7 indicates high homogeneity. In this study, the mean silhouette score was 0.7428, demonstrating strong internal consistency within each of the 10 identified clusters.

Harmonic mean of *Q* and S: The harmonic mean of the two metrics was 0.5621, providing a comprehensive evaluation of the overall clustering quality.

## 3. Results

### 3.1. Annual Publication Trend Analysis

**FIGURE 2:**
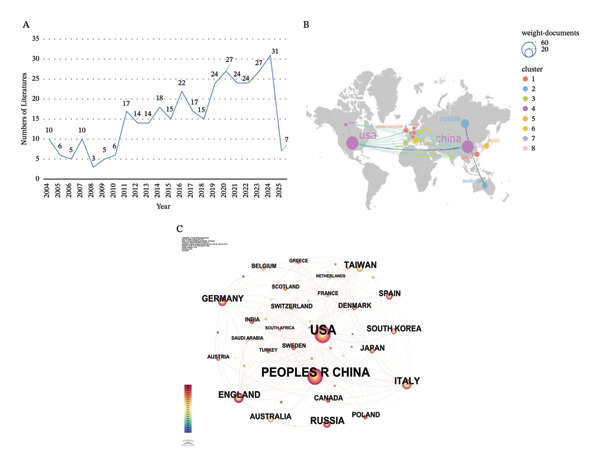
Bibliometric analysis of publication output and country regional collaboration in mtDNA and atherosclerosis research (2004–2025). (A) Annual Publication volume in the research field of mtDNA and AS; the x‐axis represents the publication year, and the y‐axis indicates the number of included articles per year, illustrating the sustained growth trajectory of the field over the 21‐year study period. (B) The geographical distribution in terms of publications. The larger circle indicates a higher number of articles, and the cooperation is exhibited as links between nodes. (C) The co‐occurrence network map of countries/regions (g‐index (k = 25), (*N* = 50) (number of network nodes), *E* = 170 (number of corrections), and density = 0.1388 (network density)).

Microsoft Excel was used to count the annual number of publications among the included 341 English publications, and relevant charts were plotted (Figure 2A). Statistics showed that the number of publications in this field has gradually increased over the years, which can be roughly divided into three development stages: The initial exploration stage (2004–2010): the annual number of publications remained at a low level of 3–10, focusing on preliminary discussions of basic mechanisms and small‐sample case correlation analysis; the fluctuating growth stage (2010–2018): the annual number of publications fluctuated between 6 and 24, with research directions concentrated on the association between mtDNA damage, oxidative stress, and AS; and the surge stage (2019–2025): this was a 6‐year period with a sharp increase in the number of publications related to mtDNA and AS, reaching a peak of 31 publications in 2024. mtDNA‐related biomarkers and intervention targets have emerged as new research hotspots.

### 3.2. Cooperation Network Analysis

#### 3.2.1. Distribution of Countries/Regions

CiteSpace software was employed to conduct a visual analysis of the cooperation among countries/regions in the research field of mtDNA and AS (corresponding to Figure 2B and C). Combined with the statistical data in Table 1, the network density (density value) of international cooperation was 0.1388, indicating relatively close academic exchanges and cooperation among countries. Regarding the distribution of countries/regions engaged in this field, the United States, China, and Russia ranked the top three in terms of publication volume (Table 1); among them, the United States and China had significantly larger nodes and served as the core participants in the academic cooperation network of this field. Meanwhile, countries such as the United Kingdom, Germany, and Japan had also formed relatively intensive cooperative connections through network links.

**TABLE 1:** Top 10 most productive countries/regions.

Rank	Country/Regions	Count	Year	Centrality
1	USA	106	2004	0.36
2	PEOPLE’S REPUBLIC OF CHINA	87	2004	0.01
3	RUSSIA	28	2012	0.11
4	ITALY	25	2005	0.08
5	ENGLAND	22	2010	0.25
6	GERMANY	19	2007	0.24
7	TAIWAN, China	18	2005	0.03
8	SOUTH KOREA	14	2004	0
9	SPAIN	13	2010	0.12
10	AUSTRALIA	13	2012	0.05

#### 3.2.2. Distribution of Institutions

Authors from 353 institutions have published research papers. The University of Texas System (centrality = 0.19) and Institute of General Pathology and Pathophysiology (centrality = 0.11) were among the most productive institutions (Table 2).

**TABLE 2:** Top 10 Institutions by number of publications in the research field of mtDNA and AS.

Rank	Institutions	Count	Centrality
1	University of Texas System	20	0.19
2	Institute of General Pathology and Pathophysiology	20	0.11
3	Russian Academy of Medical Sciences	19	0.13
4	Johns Hopkins University	19	0.09
5	National Medical Research Center of Cardiology	12	0
6	University of California System	11	0.04
7	Nanjing Medical University	11	0.03
8	Johns Hopkins Bloomberg School of Public Health	10	0.02
9	Boston University	10	0.04
10	University of Texas Health Science Center Houston	9	0.01

#### 3.2.3. Key Institutions

**FIGURE 3:**
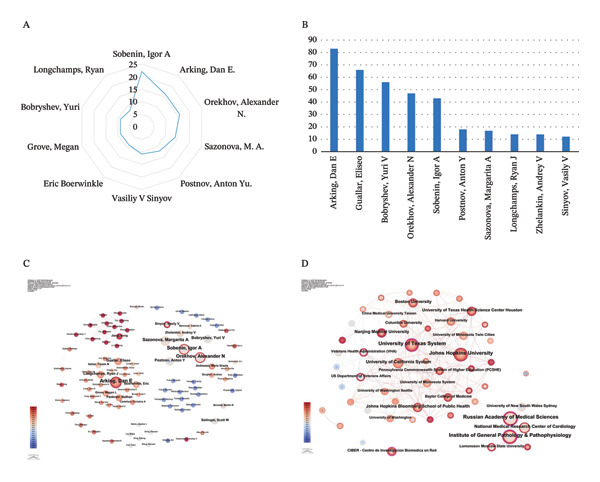
Bibliometric analysis of author and institutional contributions in mtDNA and atherosclerosis research (2004–2025). (A) Radar chart of the top 10 authors with the highest publication output. The radial axis represents the number of published articles, with the top 10 high‐yield authors labeled. (B) Bar chart of the top 10 authors ranked by H‐index, which quantifies both the quantity and quality of research outputs. (C) Author co‐occurrence map; nodes represent individual authors, with node size proportional to the number of publications; links represent collaborative co‐authorship relationships, with line thickness indicating the frequency of collaboration. The color bar at the bottom left indicates the publications year of collaborative papers (purple: 2004 and red: 2025), reflecting the temporal evolution of research teams. (D) Institutional cooperation network diagram: node size is propositional to the number of publications from the institutions; links represent interinstitutional relationships. The color bar at the bottom left corresponds to the publications year of collaborative studies (purple: 2004 and red: 2025), illustrating the temporal development of institutional research collaborations.

As shown in Figure 3D, the University of Texas System is the core leading institution in this field. Together with its associated Johns Hopkins University and University of California System, it forms the most concentrated institutional cluster for paper output. Second, the Russian Academy of Medical Sciences and the National Medical Research Center of Cardiology are the core research institutions in the Russian region. Meanwhile, Chinese institutions such as Nanjing Medical University and Nanjing Medical University Hospital have also formed active local research units. These institutions collectively constitute the core research force in this field.

#### 3.2.4. Research Trends and Geographical Distribution

From the perspective of the geographical distribution of institutions, American institutions (e.g., Johns Hopkins University and University of Texas System) have formed the largest cooperative cluster in the network map, with leading node quantity and connection density, reflecting the dominant influence of American institutions in this field. At the same time, the Russian institutional cluster (centered on the Russian Academy of Medical Sciences) and the Chinese institutional cluster (centered on relevant medical colleges and universities in Nanjing) have also formed independent regional research clusters. This distribution pattern—with the United States as the core and multiregional parallel development—reflects the research advantages of the United States in this field and also demonstrates widespread global attention to this research topic. Notably, there are relatively few cross‐regional cooperative connections between institutional clusters of different countries, indicating that transnational cooperation and exchanges in this field need to be further strengthened.

#### 3.2.5. Authors and Cocited Authors

Author cooperation network analysis (Figure 3C) reveals the core contributors and their collaboration patterns in the field. Based on the core formula of Price’s Law [16] (*M* = 0.749√Nmax), authors with ≥ 3 published papers were designated as candidates for high‐yield authors. A total of 21 core authors were identified, with the top 3 authors publishing the most papers being Sobenin, Igor A (22 papers), Arking, Dan *E* (16 papers), and Orekhov, Alexander N (16 papers) (Figure 3A). We calculated the H‐index to evaluate the quantity and quality of scholars’ research outputs. In terms of the H‐index, Arking, Dan *E*, Guallar, Eliseo, and Bobryshev, Yuri V, were far ahead. As shown in Figure 3C, the author co‐occurrence map illustrates extensive collaborations among authors as well as multiple scattered cooperation networks.

### 3.3. Keyword Analysis

#### 3.3.1. Co‐Occurrence Analysis

**FIGURE 4:**
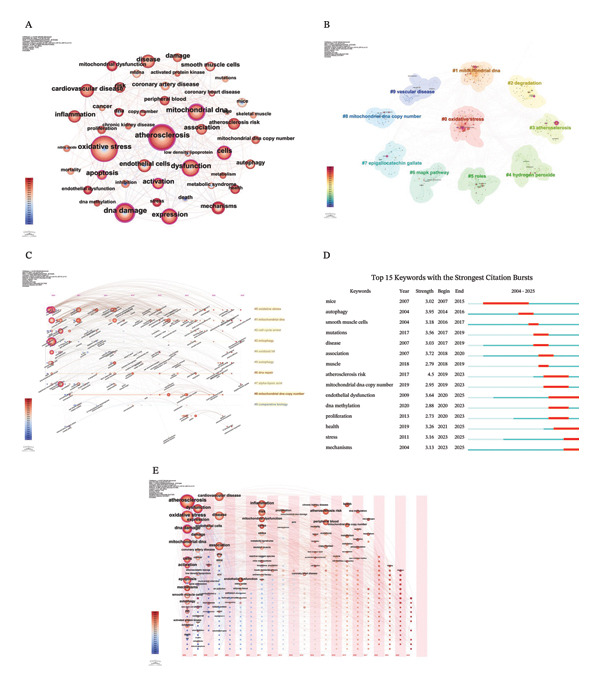
Bibliometric analysis of keywords and in mtDNA and atherosclerosis research (2004–2025). (A) Keyword co‐occurrence network map. Nodes represent keywords, with node size propositional to keyword frequency; links represent co‐occurrence relationships. The color gradient (purple to red) indicates the publications year of the keywords, reflecting the temporal evolution of research themes. (B) Cluster analysis of keywords modularity *Q* = 0.499 and mean silhouette = 0.7439, indicating significant network structure and high cluster homogeneity. Different colors represent 10 independent research clusters. (C) Timeline distributions of the top 10 clusters. The map presents the evolutionary path of research hotspots over time, showing the shift from basic mechanisms to clinical translate research. (D) Top 15 keywords with the strongest citations’ bursts; the table list keywords, their first occurrence year, burst strength, and burst period (Begin‐End). (E) Keywords timezone map of mtDNA and AS research (2004–2025). The map displays the temporal distributions and evolution of core keywords, with vertical bands representing different time periods.

Keywords are concise summaries of an article, and analyzing them can identify research hotspots and new trends. A total of 466 keywords were identified. As shown in Table 3, the top five high‐frequency keywords are AS (116 occurrences, BC = 0.25), oxidative stress (95 occurrences, BC = 0.19), dysfunction (56 occurrences, BC = 0.18), DNA damage (55 occurrences, BC = 0.22), and mtDNA (49 occurrences, BC = 0.2). This suggests a certain correlation between mtDNA dysfunction, damage, and oxidative stress. To better reflect the research hotspots and trends in this field, we used CiteSpace software to generate a high‐frequency keyword co‐occurrence network (Figure 4A) and a clustering network diagram (Figure 4B).

**TABLE 3:** Top 10 co‐occurring keywords in mtDNA and atherosclerosis research.

**Rank**		**Keywords**		**Count**	**Centrality**	**Year**

1		Atherosclerosis		116	0.25	2004
2		Oxidative stress		95	0.19	2004
3		Dysfunction		56	0.18	2005
4		DNA damage		56	0.22	2004
5		Mitochondrial DNA		49	0.2	2004
6		Expression		44	0.19	2005
7		Cells		41	0.16	2004
8		Activation		39	0.15	2004
9		Cardiovascular disease		36	0.07	2007
10		Disease		36	0.07	2007

**Rank**	**Author (year)**		**Journal**	**Key contribution**		

1	Ballinger et al. [17]		Circulation	First established the direct link between mitochondrial integrity, oxidative stress, and atherogenesis.		
2	Shimada et al. [18], Ashar et al. [19], Fidler et al. [20], Sakai et al. [21], An et al. [22]		Immunity	Demonstrated that oxidized mtDNA binds to and robustly activates the NLRP3 inflammasome during apoptosis.		
3	Yu et al. [12]		Circulation	Provided compelling evidence that mtDNA damage can promote atherosclerosis independently of reactive oxygen species (ROS).		
4	Ding et al. [10]		Sci Rep	Elucidated that ox‐LDL induces mtDNA damage, which escapes autophagy to activate TLR9‐mediated inflammation.		
5	Ashar et al. [19]		JAMA Cardiol	Landmark population cohort study proving peripheral blood mtDNA copy number as an independent predictor of CVD risk.		
6	Grebe et al. (2018)		Circ Res	Comprehensively established the crucial role of the NLRP3 inflammasome and IL‐1 pathway in atherosclerosis.		
7	Fidler et al. [20]		Nature	Revealed that the AIM2 inflammasome, sensing cytosolic DNA, exacerbates atherosclerosis in clonal hematopoiesis.		
8	Sakai et al. [21]		Circulation	Highlighted that DNA damage promotes atherosclerosis by enhancing inflammatory responses via the DNA‐sensing cGAS‐STING pathway.		
9	An et al. [22]		Int Immunopharmacol	Demonstrated that mtDNA release activates the cGAS‐STING pathway to induce endothelial cell pyroptosis.		
10	Zheng et al. [13]		Circulation	Uncovered the epigenetic regulation of mtDNA (6 mA) via METTL4, which promotes macrophage inflammation and plaque progression.		

#### 3.3.2. Clustering Analysis

As illustrated in Figure 4B, the keyword co‐occurrence network was clustered into 10 distinct thematic clusters, with a modularity *Q* score of 0.499 and a mean silhouette score of 0.7439. A modularity Q value greater than 0.3 confirms a significant and nonrandom community structure in the network, while a mean silhouette score above 0.7 demonstrates high internal homogeneity within each cluster, fully verifying the reliability and validity of the clustering results. The detailed implications of each cluster are elaborated as follows:

1. Well‐established core mechanisms: Cluster #0 oxidative stress and Cluster #1 mitochondrial DNA have been consistently studied since 2004, with high centrality scores, forming the core axis of the field. This reflects their well‐documented roles in the pathogenesis of AS, as oxidative stress–induced mtDNA damage drives proinflammatory signaling, endothelial dysfunction, and plaque progression, serving as a fundamental link between mitochondrial dysfunction and AS development.
2. Cluster #3 atherosclerosis and Cluster #9 vascular disease: As the clinical endpoint and disease spectrum of this research field, these two clusters are closely connected to the core mtDNA‐oxidative stress axis, confirming that mtDNA abnormalities are not only involved in the occurrence and progression of AS but also closely associated with the overall pathological process of vascular diseases such as coronary heart disease and ischemic stroke [23, 24].
3. Cluster #2 degradation and Cluster #4 hydrogen peroxide: These clusters further elaborate the specific molecular mechanisms of the core axis. Hydrogen peroxide, as a key ROS, directly induces mtDNA oxidative damage in vascular endothelial and smooth muscle cells [5], while the abnormal degradation of damaged mtDNA disrupts mitochondrial homeostasis, triggers inflammatory responses, and accelerates AS plaque formation, representing key regulatory nodes in mtDNA metabolism and oxidative stress pathways [10, 12].
4. Emerging mechanistic and translational insights: Cluster #6 MAPK pathway, Cluster #8 mitochondrial DNA copy number, and Cluster #7 epigallocatechin gallate (EGCG) represent important research directions in recent years. The MAPK signaling pathway is a key downstream cascade activated by mtDNA damage, mediating vascular smooth muscle cell proliferation and inflammatory activation [25]; mtDNA copy number (mtDNA‐CN) has become a core noninvasive biomarker for AS risk assessment, with reduced levels independently associated with increased coronary artery disease risk [26, 27]; while EGCG, as a natural active component, exerts anti‐AS effects by protecting mtDNA and inhibiting oxidative stress, highlighting the translational potential of traditional Chinese medicine and natural products in this field [28, 29].
5. Cluster #5 roles, as a general thematic cluster, focuses on the functional exploration of mtDNA in AS and has a weak independent correlation with the core axis, which reflects the basic research orientation of continuously expanding the multidimensional roles of mtDNA in AS pathogenesis, without affecting the identification of the core research framework of the field.

#### 3.3.3. Timeline Analysis

The timeline plot (Figure 4C) illustrates the evolutionary trajectory of mtDNA and AS research from 2004 to 2025: the field started with foundational investigations centered on oxidative stress and mtDNA damage (2004–2010), then expanded to mechanistic studies on hydrogen peroxide, MAPK pathway, and mtDNA degradation (2011–2018), and in recent years has focused on translational research including mtDNA‐CN biomarker and natural product (EGCG) intervention (2019–2025), showing a clear evolution from basic mechanism to clinical translation.

#### 3.3.4. Timezone Diagram Analysis

The keyword timezone map (Figure 4D) visually presents the temporal evolution of research themes. Keywords including AS, oxidative stress, mtDNA span all timezones with large node sizes, confirming their status as long‐term core themes. In the early stage (2004–2010), research focused on basic pathological mechanisms; in the middle stage (2011–2018), molecular pathways such as MAPK and mtDNA degradation became new hotspots; and in the recent stage (2019–2025), mtDNA‐CN, EGCG and vascular disease emerged intensively, reflecting a shift from basic research to clinical biomarker screening and targeted intervention research.

### 3.4. Keywords Burst Analysis

By analyzing the burst keywords in the Publications (Figure 4E), it was found that the field has distinct research hotspots in different periods. From 2004 to 2015, basic keywords such as mice (strength = 3.02) dominated, focusing on verifying mtDNA damage and AS correlation through cellular and animal models. From 2011 to 2019, burst keywords including autophagy (strength = 3.95) and mutations (strength = 3.56) emerged, marking the deepening of molecular mechanism research. Since 2019, the research has entered the clinical translation phase, with core burst keywords including AS risk, mtDNA‐CN, DNA methylation, and the continuous burst of endothelial dysfunction (strength = 3.64). These results indicate that the field is gradually moving toward clinical risk assessment, epigenetic regulation, and targeted intervention, laying a foundation for the clinical transformation of mtDNA‐targeted therapies.

### 3.5. Latent Dirichlet Allocation (LDA) Topic Modeling and Methodological Triangulation

To overcome the limitations of predefined keyword co‐occurrence and further reveal the latent semantic architecture of the field, a LDA generative probabilistic model was applied to the titles and abstracts of the included articles. The LDA algorithm successfully identified three dominant latent topics, which strongly corroborate the clustering results and highlight the core intellectual trajectories of mtDNA in AS research.

#### 3.5.1. Topic 1: mtDNA‐CN as a Predictive Biomarker for Cardiovascular Risk

This topic encompasses the extensive epidemiological and clinical focus on mtDNA‐CN in peripheral blood leukocytes. Large‐scale cohort studies have established that reduced mtDNA‐CN serves as an independent, noninvasive biomarker for mitochondrial dysfunction, correlating significantly with incident CVD and primary risk stratification. Furthermore, genomic approaches are frequently utilized within this topic to explore the causal pathways between lipid metabolism and mtDNA‐CN fluctuations.

#### 3.5.2. Topic 2: mtDNA Somatic Mutations, Heteroplasmy, and Clonal Expansion

The second latent topic highlights the genetic and mutational landscape of mtDNA in atherogenesis. Publications within this cluster discuss how specific atherogenic mtDNA somatic mutations and varying levels of heteroplasmy accumulate within the arterial wall and circulating immune cells. These genetic alterations compromise mitochondrial respiratory chain integrity and cellular bioenergetics, acting as an internal trigger for plaque progression. Additionally, recent landmark studies link these mutations to clonal hematopoiesis, which exacerbates AS via inflammasome activation.

#### 3.5.3. Topic 3: mtDNA Damage–Induced Inflammatory Signaling and the Microenvironment

The third topic delves into the mechanistic pathways linking damaged mtDNA to vascular inflammation. When mitochondrial integrity is compromised, damaged mtDNA escapes into the cytoplasm, acting as a Damage‐Associated Molecular Pattern (DAMP). This cytosolic mtDNA is recognized by innate immune sensors, notably triggering the cGAS‐STING pathway and TLR9 signaling. These pathways subsequently activate downstream inflammatory cascades and apoptosis in endothelial cells and macrophages. Furthermore, emerging epigenetic studies indicate that METTL4‐mediated mtDNA N6‐methyldeoxyadenosine (6 mA) modifications play a critical role in promoting macrophage inflammation and plaque instability.

#### 3.5.4. Methodological Triangulation

The latent topics extracted via the unsupervised LDA model exhibit high congruence with the keyword clusters generated by CiteSpace (e.g., Cluster #0 oxidative stress and Cluster #8 mtDNA copy number). This methodological triangulation confirms the robustness of our bibliometric findings, demonstrating that the field has decisively transitioned from phenomenological observations of oxidative damage to the precise mechanistic elucidation of mtDNA‐triggered immune sensing and its clinical application as a biomarker.

### 3.6. Core Landmark Studies and Intellectual Base

The intellectual base of this field has evolved through four critical paradigm shifts driven by these highly cited landmark papers.

#### 3.6.1. Phase 1: Early Discovery and mtDNA Damage

Exploratory research was largely anchored by Ballinger et al. [17], who first demonstrated that mtDNA damage is profoundly correlated with the extent of atherogenesis. A major leap in understanding occurred when Yu et al. (2013) demonstrated that mtDNA damage can promote AS independently of ROS through its profound effects on smooth muscle cells and monocytes, linking it directly to higher‐risk plaques.

#### 3.6.2. Phase 2: DAMPs, Autophagy, and Innate Immune Sensing

As research deepened into lipid metabolism and mitochondrial dysfunction, Ding et al. [10] mapped a crucial pathogenic axis, revealing that ox‐LDL induces extensive mtDNA damage. Damaged mtDNA that escapes autophagic clearance acts as a DAMP, directly activating the TLR9 inflammatory signal. Concurrently, Shimada et al. [18] provided highly cited evidence that oxidized mtDNA is a primary ligand that activates the NLRP3 inflammasome.

#### 3.6.3. Phase 3: Advanced Cytosolic DNA‐Sensing Pathways

In recent years, the frontier has rapidly advanced toward sophisticated cytosolic nucleic acid sensors. Landmark studies, such as those by Sakai et al. [21] and An et al. [22], have elucidated how cytosolic mtDNA triggers the cGAS‐STING pathway, driving endothelial pyroptosis and plaque vulnerability. Similarly, Fidler et al. [20] identified the pivotal role of the AIM2 inflammasome in exacerbating AS during clonal hematopoiesis. Furthermore, cutting‐edge studies like Zheng et al. [13] have introduced mitoepigenetics, showing that METTL4‐mediated mtDNA N6‐methyldeoxyadenosine (6 mA) promotes macrophage inflammation.

#### 3.6.4. Phase 4: Clinical Translation

On the clinical front, Ashar et al. [19] successfully translated these bench findings to population science, establishing that a reduced mtDNA‐CN in circulating leukocytes serves as a robust, noninvasive biomarker for primary CVD risk stratification. Together, these top 10 core publications form the structural backbone of the evolving mtDNA–AS knowledge graph.

## 4. Discussion

### 4.1. Overview of Research Trends

The bibliometric analysis of publications from 2004 to 2025 reveals a steady and robust expansion in the field of mtDNA and AS. Through the utilization of CiteSpace, our study mapped the knowledge domains, capturing the evolutionary trajectory from foundational studies focusing on oxidative damage to the current mechanistic investigations of innate immune sensing. The transition observed in keyword clusters suggests a trend toward deeper molecular precision and the exploring of noninvasive biomarkers.

### 4.2. Core Pathogenic Mechanisms: From Oxidative Stress to Innate Immune Sensing

Our keyword clustering and LDA topic modeling indicate that the core mechanisms of mtDNA in atherogenesis have become increasingly well defined. Early landmark studies, such as the seminal work by Ballinger et al. [17], firmly established that mitochondrial integrity and mtDNA oxidative damage are closely associated with the extent of atherogenesis. Subsequently, a critical advancement was provided by Yu et al. [12], who demonstrated that mtDNA damage could promote AS independently of ROS by profoundly affecting smooth muscle cells and monocytes, thereby correlating with higher‐risk plaques.

In recent years, the interplay between lipid metabolism, impaired mitophagy, and innate immune sensing has emerged as a dominant theme. As shown in our bibliometric network, ox‐LDL induces extensive mtDNA damage. When this damaged mtDNA escapes autophagic clearance, it acts as a highly immunogenic DAMP. Once released into the cytosol or extracellular space, mtDNA is recognized by innate immune sensors. For instance, it can robustly activate TLR9‐mediated inflammatory responses, which exacerbate the plaque microenvironment. Concurrently, recent publications heavily highlight the activation of the cGAS‐STING pathway by cytosolic mtDNA, which drives endothelial cell pyroptosis and promotes plaque vulnerability. Other inflammasomes, notably the AIM2 inflammasome, have also been identified as crucial components that exacerbate AS, particularly in conditions like clonal hematopoiesis. Furthermore, emerging evidence has unveiled novel epigenetic regulatory pathways; for example, Zheng et al. recently demonstrated that METTL4‐mediated mtDNA N6‐methyldeoxyadenosine (6 mA) promotes macrophage inflammation, revealing an additional layer of mitoepigenetic control in AS.

### 4.3. Emerging Technologies and Cautious Future Directions

While our bibliometric results primarily reflect established hotspots, the broader scientific publications point toward several promising future directions. Topics such as extracellular vesicles (EVs) as potential carriers for intercellular mtDNA crosstalk, the application of single‐cell RNA sequencing, and spatial transcriptomics are garnering significant interest. These advanced omics technologies hold the theoretical potential to dissect the complex cellular heterogeneity of mtDNA mutations within atherosclerotic lesions.

However, it must be discussed cautiously: these topics are currently in the early exploratory stages and are not yet clearly supported as dominant, self‐sustaining clusters in our current bibliometric network. Their application in the specific context of mtDNA and AS remains limited, and substantial further validation—particularly through robust in vivo models and large‐scale human cohorts—is required before they can be considered mainstream research paradigms or utilized to draw definitive mechanistic conclusions. Therefore, these emerging technologies are best viewed as future methodological opportunities rather than established scientific trajectories.

### 4.4. Clinical Translation and Biomarker Potential

Another significant trend identified in our analysis is the investigation of mtDNA‐CN in peripheral blood. Landmark population–based cohort studies, such as the one conducted by Ashar et al. [19], have provided compelling epidemiological evidence that reduced mtDNA‐CN in circulating leukocytes is independently associated with an increased risk of incident CVD.

While these bibliometric trends suggest a growing interest in utilizing mtDNA‐CN as a noninvasive predictive biomarker, we must emphasize that current findings do not directly prove clinical maturity or translational readiness. The transition from epidemiological associations to standardized clinical application faces numerous hurdles. Standardized measurement protocols, validation across diverse genetic ancestries, and large‐scale, multicenter prospective trials are still needed. Consequently, while the concept of mtDNA‐targeted diagnostics and therapies holds immense promise, its integration into routine clinical practice should be viewed as a long‐term goal that requires rigorous and continuous evaluation.

## 5. Conclusion

This bibliometric analysis provides a comprehensive, data‐driven overview of 21 years of research on mtDNA and AS, revealing a field that has evolved from foundational mechanistic studies to a focus on translational and clinical applications. Our findings highlight the central role of mtDNA‐mediated oxidative stress and inflammation in atherogenesis, while also identifying emerging frontiers such as EV‐mediated mtDNA transfer and precision medicine approaches.

By distinguishing well‐established topics from genuinely emerging directions, this study offers a roadmap for future research priorities. The correlation between bibliometric hotspots and key scientific advances underscores the potential of these methods to inform strategic investment and accelerate progress. While limitations exist, particularly regarding language and database constraints, our sensitivity analyses confirm the robustness of our core conclusions.

Ultimately, this work underscores the critical need for continued investment in mtDNA‐related AS research, with a focus on translating mechanistic insights into novel biomarkers and targeted therapies. By doing so, we can move closer to reducing the global burden of this devastating disease.

## Author Contributions

Yun Ouyang: conceptualization, formal analysis, methodology, supervision, writing–original draft, and writing–review and editing. Ming Zhang: data curation, software, visualization, and writing–original draft. Gesheng Wang: funding acquisition, methodology, resources, supervision, and writing–review and editing. Puxuan Min and Mengxiong Guo: writing–review and editing.

## Funding

The authors declare that financial support was received for the research, authorship, and/or publication of this article. This work was supported by the National Natural Science Foundation of China (NSFC) Supported Project (81874422).

## Conflicts of Interest

The authors declare no conflicts of interest.

## Data Availability

The data that support the findings of this study are openly available in web of science at https://webofscience.clarivate.cn/wos/woscc/smart-search.
